# Characterization of NO-Induced Nitrosative Status in Human Placenta from Pregnant Women with Gestational Diabetes Mellitus

**DOI:** 10.1155/2017/5629341

**Published:** 2017-03-16

**Authors:** Francisco Visiedo, Celeste Santos-Rosendo, Rosa M. Mateos-Bernal, M. del Mar Gil-Sánchez, Fernando Bugatto, Manuel Aguilar-Diosdado, Carmen Segundo, Cristina López-Tinoco

**Affiliations:** ^1^Research Unit, Puerta del Mar University Hospital, Cádiz, Spain; ^2^Department of Genetic, Faculty of Biology, University of Seville, Seville, Spain; ^3^Department of Obstetrics and Gynecology, Puerta del Mar University Hospital, Cádiz, Spain; ^4^Department of Endocrinology and Nutrition, Puerta del Mar University Hospital, Cádiz, Spain; ^5^Salus Infirmorum, Faculty of Nursing, University of Cádiz, Cádiz, Spain

## Abstract

Dysregulation of NO production is implicated in pregnancy-related diseases, including gestational diabetes mellitus (GDM). The role of NO and its placental targets in GDM pregnancies has yet to be determined. S-Nitrosylation is the NO-derived posttranslational protein modification that can modulate biological functions by forming NO-derived complexes with longer half-life, termed S-nitrosothiol (SNO). Our aim was to examine the presence of endogenous S-nitrosylated proteins in cysteine residues in relation to antioxidant defense, apoptosis, and cellular signal transduction in placental tissue from control (*n* = 8) and GDM (*n* = 8) pregnancies. S-Nitrosylation was measured using the biotin-switch assay, while the expression and protein activity were assessed by immunoblotting and colorimetric methods, respectively. Results indicated that catalase and peroxiredoxin nitrosylation levels were greater in GDM placentas, and that was accompanied by reduced catalase activity. S-Nitrosylation of ERK1/2 and AKT was increased in GDM placentas, and their activities were inhibited. Activities of caspase-3 and caspase-9 were increased, with the latter also showing diminished nitrosylation levels. These findings suggest that S-nitrosylation is a little-known, but critical, mechanism by which NO directly modulates key placental proteins in women with GDM and, as a consequence, maternal and fetal anomalies during pregnancy can occur.

## 1. Introduction

Gestational diabetes mellitus (GDM) is a glucose intolerance of varying severity with onset at, or first recognition during, pregnancy and prevalence of around 5% of all pregnancies [1]. GDM increases perinatal morbidity and mortality as well as subsequent diabetes mellitus type 2 (DMT2) in the mother [2]. The pathophysiology of GDM remains unclear, although oxidative/nitrosative stress induced by NO and oxygen radical presence in addition to proinflammatory status have been reported as key factors.

S-Nitrosylation is a well-documented mechanism of NO-induced protein modification, which is related with modifications of processes such as cell proliferation, cell survival, and apoptosis [3, 4].

A common feature of pregnancy is inflammation, which is aggravated by obesity and GDM. One of the main features in the inflammatory response is NO production, which causes molecular damage in a process termed nitrosative stress. This feature, in addition to maternal hyperglycemia and altered cytokine profile, constitutes a main set of pathogenic factors that induce measurable alterations in GDM placenta [5]. These factors are associated with an increased risk of adverse perinatal outcomes and metabolic diseases in the mother as well as in the offspring later in life [6, 7]. These changes occur alongside those of normal pregnancy which is a high-energy demanded state characterized by high utilization of oxygen. Both these features lead to increased oxidative stress. Also, changes in the prooxidant and antioxidant defenses are implicit in the pregnancy process [8].

In pathological situations such as GDM, oxidative as well as nitrosative stress can exert synergistic effects. For example, S-nitrosylation has been reported as a possible chemical process through which antioxidant enzyme activity is regulated [9]. Structural alterations in GDM full-term placentas have been described. These include increased villous immaturity and increased angiogenesis [10]. Also, molecules involved in placental function are altered in GDM, for example, ERK1/2 and AKT/PI3-K signaling pathways in trophoblast proliferation, and differentiation processes are affected in GDM [11].

Further, an altered balance between cell proliferation and apoptosis can be observed as part of GDM pathogenic process which, potentially, can result in larger placenta size [12]. Caspase activity alteration (oxidative or nitrosative stress-mediated) could explain this phenomenon [4].

We investigated the presence of NO-mediated nitrosative modifications of proteins in human placenta from women with GDM. We compared the findings in control individuals with the hypothesis that placental nitrosative status is significantly altered in GDM pregnancies. This alteration may be verified via the detection of S-nitrosothiol (SNO) groups in proteins related to antioxidant defense, cell survival, and apoptosis.

## 2. Methods

### 2.1. Reagents and Antibodies

All chemical reagents required for this study were obtained from Sigma (St. Louis, MO, USA) and Bio-Rad (Hercules, CA, USA). Antibodies (anti-p-ERK1,2, anti-ERK total, anti-caspase 3, anti-peroxiredoxin-1, anti-catalase, and *β*-actin) were purchased from Cell Signaling Technology (Danvers, MA, USA), anti-caspase 9 was purchased from MBL (Nagoya, Japan), and iNOS was purchased from R&D Systems (Minneapolis, MN, USA).

### 2.2. Study Population and Tissue Collection

This study was performed on placentas from control and GDM pregnancies. GDM was diagnosed according to the criteria of the National Diabetes Data Group [13] and validated by the* Grupo Español de Diabetes y Embarazo* (Spanish Group of Diabetes and Pregnancy) [14]. Informed consent was obtained from all patients in accordance with the* Hospital Universitario Puerta del Mar* (HUPM) Ethics Committee requirements and those of the Declaration of Helsinki. Full-term placentas were collected immediately after elective Cesarean section at the Department of Obstetrics and Gynecology,* HUPM* (Cádiz). The indications for elective caesarean section at term were breech presentation,* placenta previa*, and a previous Caesarean section. None of the pregnant women recruited into this study had history of chronic diseases, hypertension, preeclampsia, fetal anomalies, smoking habit, and infections.

Placentas were placed on ice and processed within 10 min of delivery. Decidua tissue and large vessels were removed from villous placenta by blunt dissection under aseptic culture conditions. Small fragments (~100 mg wet wt.) were removed from the central region of the placenta near the umbilical cord, rinsed in cold PBS to eliminate attached blood, and stored at −80°C until being required for analyses.

### 2.3. SDS-Western Blotting for the Measurement of Total Protein Expression

Frozen placenta samples were thawed and homogenized in lysis buffer containing 20 mM Tris-HCl (pH 7.5), 150 mM NaCl, 1 mM Na_2_EDTA, 1 mM EGTA, 1% Triton, 2.5 mM sodium pyrophosphate, 1 mM beta-glycerophosphate, 1 mM Na_3_VO_4_, 2 *μ*M leupeptin plus 1 mM phenylmethylsulfonyl fluoride (Sigma), and protease inhibitors (Protease Inhibitor Cocktail, Sigma). After 20 minutes on ice, the homogenates were sonicated and centrifuged at 11000 ×g for 5 minutes at 4°C. Quantification of protein in the supernatant was determined using the bicinchoninic acid (BCA) assay (Thermo Scientific, Madrid, Spain).

Protein extracts (40 *µ*g/sample) were heat-denatured at 100°C for 5 minutes in Laemmli sample buffer containing 0.5 M Tris (pH 6), 10% SDS, 20% glycerol, and 0.5% (w/v) bromophenol blue dye. Samples of the protein extracts were separated on 10% SDS-PAGE, transferred to polyvinylidene fluoride membrane, and blotted with antibodies directed against the proteins under study (diluted at 1 : 500–1 : 1000). Secondary antibodies were used at a dilution of 1 : 5000. Signals were detected by chemiluminescence (Immun-Star Kit, Bio-Rad) and band densitometry was quantified with ImageJ software.

### 2.4. Detection of S-Nitrosylated Proteins Using the Biotin-Switch Technique

The biotin-switch assay was performed as described by Jaffrey and Snyder with a few modifications [15]. Essentially, placental tissue was homogenized with HEN buffer (100 mM HEPES-NaOH, pH 7.2, 1 mM EDTA, and 0.1 mM neocuproine pH 7.2) containing 1% Triton X-100 and 0.1% SDS. The protein concentration was determined using the BCA protein assay (see above). An aliquot of 40 *μ*g protein was mixed with 400 *μ*L blocking buffer (25% SDS and 0.1% MMTS in HEN buffer) at 55°C for 30 minutes in the dark with agitation to enable S-methylthiolation of each cysteine thiol with S-methyl methanethiosulfonate (MMTS). After blocking, the extracts were precipitated with cold acetone and resuspended in 100 *μ*L HENS buffer (HEN plus 1% SDS) to prevent biotinylation of primary amines. Thiol groups were then reduced with 50 mM sodium ascorbate (Sigma) and biotinylated with 33 *μ*L reducing buffer (4 mM Biotin-HPDP; Pierce, Waltham, MA, USA) for 1 h at room temperature. Proteins were reprecipitated using acetone and resuspended in HNES buffer. For purification of biotinylated proteins, samples were diluted with neutralizing buffer (20 mM HEPES-NaOH, pH 7.4, 100 mM NaCl, 1 mM EDTA, and 0.5% Triton X-100). The proteins were drawn out of suspension using NeutrAvidin Agarose resin beads (Thermo Scientific) and incubated overnight at 4°C. Finally, the beads were washed (×3) with neutralizing buffer and proteins were eluted with SDS sample buffer for 5 min at 95°C and subjected to Western blot analysis.

### 2.5. Enzyme Activity Determinations

Catalase activity was determined by spectrophotometric absorbance at 240 nm of hydrogen peroxide breakdown, as described in the method by Aebi [16].

### 2.6. Proliferation Assays

10 *μ*m cryosections from placenta were fixed with acetone 100% for 15 min at 4°C, washed with PBS, and incubated for 30 min with 0.1% Triton X-100 in PBS for tissue permeabilization and incubated for 30 min with 4% BSA blocking solution in PBS at room temperature. Double immunostaining was performed overnight at 4°C using polyclonal rabbit anti-Ki-67 (Abcam, Cambridge CB4 OFL, UK) and monoclonal mouse cytotrophoblast specific anti-desmosomal protein (Sigma-Aldrich, St. Louis, MO, USA) antibodies according to the manufacturers' instructions. Antibody staining was revealed using anti-rabbit IgG Alexa 488 conjugated and anti-mouse IgG Alexa 546 conjugated antibodies (Molecular Probes, Inc., Eugene, OR, USA). Double positive cells and placenta areas were quantified and results were expressed as number of desmosome^+^/Ki-67^+^ cells/mm^2^ placenta.

### 2.7. Detection of Apoptosis

Cytotrophoblast apoptosis was determined using the DeadEnd Fluorometric TUNEL System (Promega, Madison, WI, USA) according to the manufacturer's instructions, simultaneously with cytotrophoblast immunostaining (using monoclonal mouse anti-desmosomal protein; Sigma-Aldrich, St. Louis, MO, USA). Quantification was as above, and the results were expressed as number of desm^+^/TUNEL^+^ cells/mm^2^ of placenta.

### 2.8. Statistical Analysis

The significance tests used were the Mann–Whitney *U* test and Student's* t*-test. *p* < 0.05 was accepted as a significant difference between variables compared.

## 3. Results 

### 3.1. Clinical and Anthropometric Characteristics of the Pregnant Participants

As shown in Table 1, control and GDM groups had similar anthropometric variables, except for significantly higher maternal pregestational body mass index (BMI) in GDM versus control group (*p* = 0.001).

### 3.2. iNOS Expression in GDM Placenta

**Figure 1:**
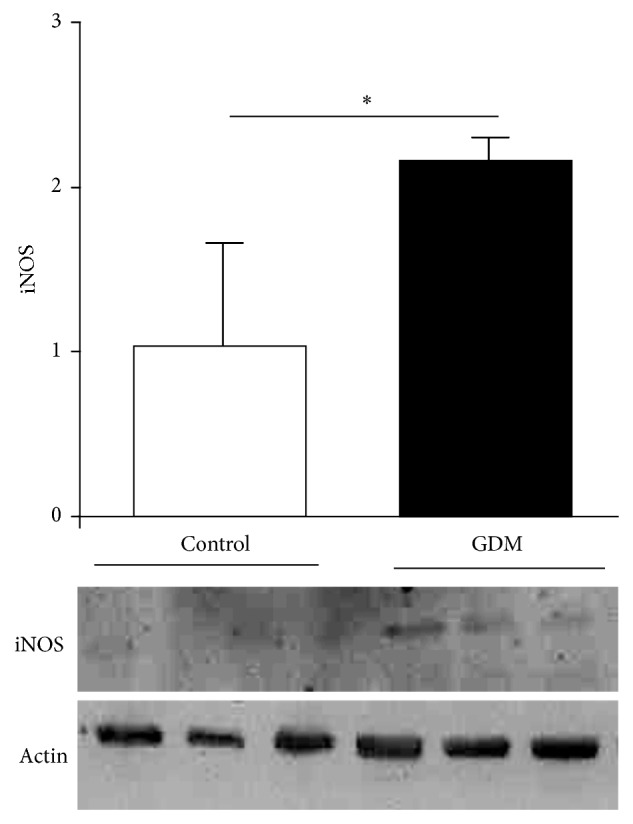
iNOS expression in GDM.* Notes*. iNOS expression quantified by Western blot of placental tissue obtained from control and GDM pregnancies. Results are expressed as means ± SEM of iNOS to actin ratio measured by densitometry (*n* = 3). Lower panel is a representative image from an experiment. Units on bar chart *y*-axis are arbitrary. ^*∗*^*p* < 0.05, GDM versus control.

iNOS expression in placental tissue from the GDM group compared to control (depicted in Figure 1) shows an increment in iNOS expression in placenta from GDM demonstrating that inducible isoform of NOS is activated under pathological conditions.

### 3.3. Identification of Placenta S-Nitrosylated Target Enzymes Related to Antioxidant Defense

**Figure 2:**
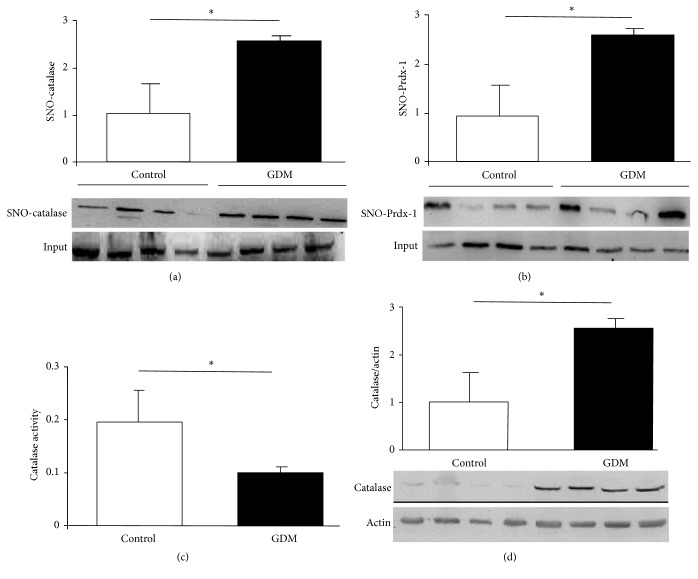
S-Nitrosylation in antioxidant enzymes.* Notes*. ((a) and (b)) S-Nitrosylation measured in placental tissue obtained from control and GDM pregnancies. Methods used were biotin-switch technique for catalase (a) and peroxiredoxin-1 (b). Results in bar charts are mean ± SEM of SNO-protein (*n* = 6) relative to input (total quantity of studied protein present in each sample). Units on bar chart *y*-axes are arbitrary. Representative images of immunoblotted SNO-proteins are shown under each graph. Catalase activity (c) and expression (d) were quantified by spectrophotometric analysis and immunoblotting, respectively. Placental tissues were obtained from control and GDM pregnancies. Results reported as mean ± SEM of enzymatic activity (*n* = 8) and catalase to actin ratio measured by densitometry (*n* = 3); lower panel is a representative image of immunoblotted proteins. ^*∗*^*p* < 0.05, GDM versus control.

Protein S-nitrosylation is a reversible posttranslational modification that regulates the function of many target proteins (including enzymes) via the action of nitric oxide (NO). To assess whether full-term placentas from women with GDM had a characteristic nitrosative profile of enzymes associated with protection against oxidative stress-induced cell death, overall levels of NO-mediated S-nitrosylation were measured as covalent attachments to cysteine residues in the main antioxidant proteins. Increased levels of S-nitrosylation were exhibited by catalase (Figure 2(a)) and peroxiredoxin (detoxifying enzymes of hydrogen peroxide; Figure 2(b)) in GDM placentas compared to the control group. S-Nitrosylation levels of other antioxidant enzymes such as Cu/Zn-superoxide dismutase (Cu/Zn-SOD-1) and glutathione peroxidase (GPx-1) remained unchanged (data not shown). With regard to catalase, total amount of protein and enzymatic activity were determined. An increment of catalase total amount (Figure 2(c)) accompanied by an inhibition of its activity (Figure 2(d)) was observed in placentas from women with GDM in comparison with control group. These observations suggest that NO-induced S-nitrosylation inhibits enzymatic activity of key endogenous antioxidants that have a protective function against oxidative stress in placenta in pregnant women with GDM.

### 3.4. Identification of Placental S-Nitrosylated Target Enzymes Related to Cell Survival

**Figure 3:**
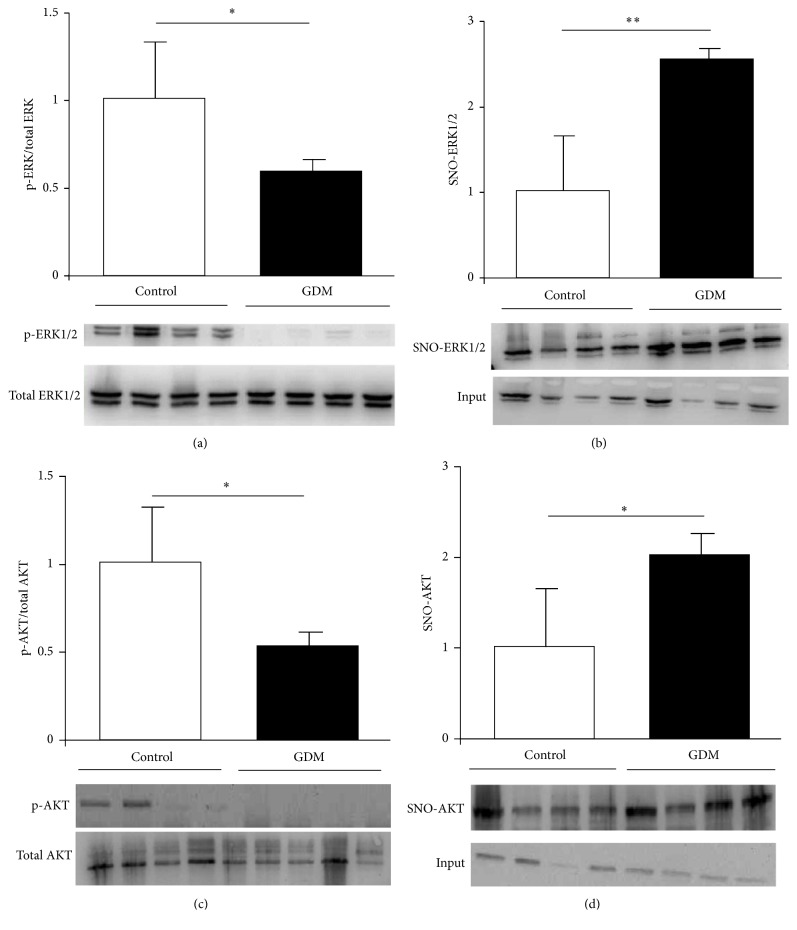
S-Nitrosylation and activation level in AKT and ERK1/2.* Notes*. ((a) and (c)) ERK1/2 (a) and AKT (c) phosphorylation quantified by Western blotting in control and GDM placental tissue. Results are expressed as means ± SEM of phosphoprotein to total-protein ratio measured by densitometry (*n* = 6). Lower panels are representative images of immunoblotted proteins. ((b) and (d)) S-Nitrosylation measured in placental tissue obtained from control and GDM pregnancies. Methods used were biotin-switch technique in ERK1/2 (b) and AKT (d). Results are presented in bar charts as mean ± SEM of SNO-protein (*n* = 6). Lower panels are representative images of immunoblotted SNO-proteins. Units on *y*-axes of bar charts are arbitrary. ^*∗*^*p* < 0.05, GDM versus control; ^*∗∗*^*p* < 0.01, GDM versus control.

Activation of cell survival-associated ERK1/2 pathway is regulated by mechanisms related to posttranslational modifications, including downstream scaffolds and phosphorylation of threonine and tyrosine residues. Hence, we quantified the level of placental ERK1/2 phosphorylation in control and GDM pregnancies. As depicted in Figure 3(a), phosphorylated ERK1/2 decreased significantly in GDM placentas compared to control placentas. Moreover, the levels of placental ERK1/2-SNO were measured in both groups by biotin-switch assay, and a significant increase was found in placental tissue from pregnant women with GDM (Figure 3(b)).

AKT is another signaling pathway involved in placental function and the main proteins of the pathway are also regulated by posttranslational modifications. We quantified AKT phosphorylation in GDM and control placental tissue and observed a significant increase in GDM (Figure 3(c)). In addition, AKT S-nitrosylation was determined and the results showed that the GDM group had higher levels of this posttranslational modification than control group (Figure 3(d)).

These results highlight S-nitrosylation of ERK1/2 and AKT as crucial mechanisms by which NO directly regulates proteins linked to several biological pathways associated with cell survival and function in placental tissue during pregnancy.

### 3.5. Determination of Cytotrophoblast Proliferation

**Figure 4:**
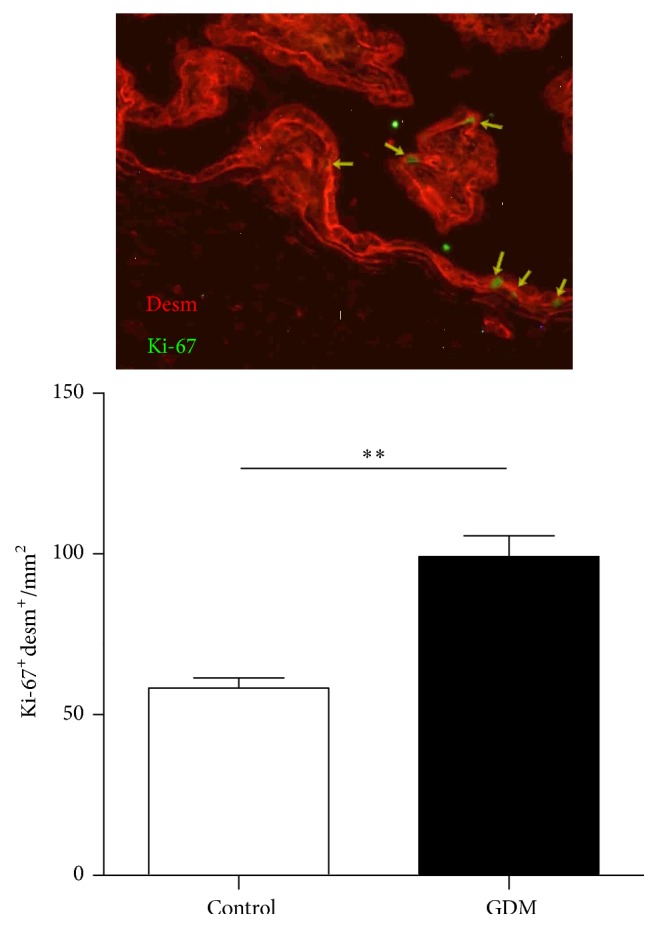
Proliferation level in trophoblasts.* Notes*. Cytotrophoblast proliferation quantified by immunohistochemistry in control and GDM placental tissue. Results are expressed as means ± SEM of desmosome protein (desm) and Ki-67 positive cells per area (mm^2^) (*n* = 7). Upper panel is a representative image of desmosome protein (red) and Ki-67 (green) immunostaining. Units on *y*-axes of bar charts are arbitrary. ^*∗∗*^*p* < 0.01, GDM versus control.

GDM-induced alteration of above-described enzyme activities can be related with a disrupted balance between apoptosis and proliferation in placental cytotrophoblast. We quantified proliferating cytotrophoblasts in placental tissue from pregnant women with GDM and observed a significant increase in GDM placentas compared with control group (Figure 4).

### 3.6. Apoptosis Quantification and Identification of Placental S-Nitrosylated Caspases

**Figure 5:**
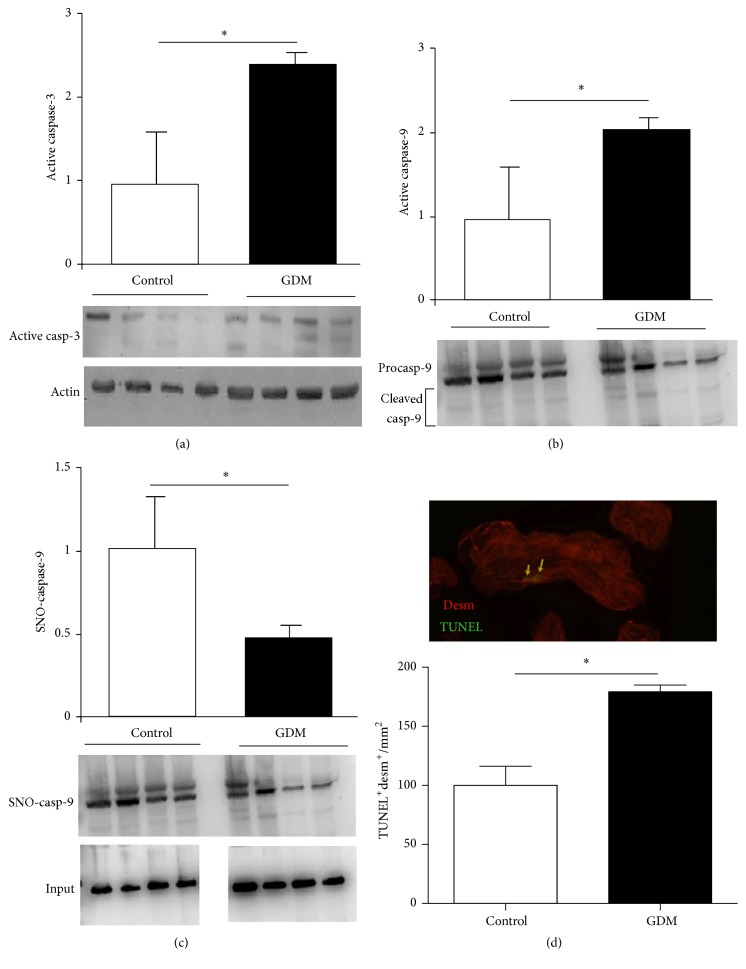
S-Nitrosylation and activation level in caspases.* Notes*. ((a) and (b)) Active fragment of caspase-3 (a) and caspase-9 (b) determined in control and GDM placental tissue by Western blotting. Results are expressed as means ± SEM of active caspases to actin ratio measured by densitometry (*n* = 3). Lower panels are representative images of immunoblotted proteins. (c) Caspase-9 S-nitrosylation measured in both experimental groups by biotin-switch technique. Results are presented in bar charts as mean ± SEM of SNO-caspase-9 (*n* = 6). Lower panel is a representative image of experiment. (d) Cytotrophoblast apoptosis quantified by TUNEL technique in control and GDM placental tissue. Results are expressed as means ± SEM of desmosome protein (desm) and TUNEL-positive cells per area (mm^2^) (*n* = 7). Upper panel is a representative image of desmosome protein (red) and TUNEL (green) immunostaining. Units on graph *y*-axis are arbitrary. ^*∗*^*p* < 0.05, GDM versus control.

NO can regulate this programmed cell death via S-nitrosylation of active caspases. We analyzed caspase-9 and caspase-3 activation and observed an increment in placental tissue from pregnant women with GDM (Figures 5(a) and 5(b)). S-Nitrosylation was measured to evaluate the regulatory effect of NO effect on caspase-9, and the results showed a significant decrease in caspase-9 SNO levels in GDM placentas relative to the control group (Figure 5(c)).

In addition, cytotrophoblast apoptosis was higher in GDM placental tissue than in control (Figure 5(d)).

## 4. Discussion

In the present study, we highlight that NO-mediated nitrosative status is altered in placental tissue from GDM pregnancies. Our findings are based on the direct evaluation of SNO-proteins involved in various cellular physiological processes including antioxidant defense, apoptosis, and cellular signaling transduction, all of which combine in providing a mechanism for redox-based regulation of standard placental function.

NO is a pleiotropic signaling molecule which is synthesized by the enzyme NOS and regulates essential cellular processes such as muscle relaxation and blood pressure regulation [17]. Overproduction of NO by aberrant iNOS induction is implicated in the pathogenesis of many disorders including neurodegenerative diseases, inflammatory and autoimmune diseases, cardiovascular diseases, and cancer [18]. In the case of GDM, inflammatory status and maternal hyperglycemia are involved in the generation of nitrosative stress resulting from induction of* iNOS* gene expression [19]. This is in addition to the described increased NO during normal pregnancy [20]. The principal target of these alterations is the placenta.

Posttranslational protein modifications are some of mechanisms by which NO alters signaling pathways leading to pathologic events. Recently, protein S-nitrosylation has been recognized as an important, reversible, posttranslational modification consisting of a covalent attachment of a nitrogen monoxide group to the thiol side-chain of cysteine. Under nitrosative stress conditions, S-nitrosylation can result in altered protein activity and adverse biological consequences [21].

Our data (Figure 1) demonstrate that GDM causes increased iNOS expression in full-term placental tissue, which is in accordance with other reports [22]. Of note is the fact that we had used only placentas obtained from elective cesarean deliveries so as to rule out potential effects of labor on placental oxidative and nitrosative stress. Frequently, oxidative/nitrosative stress occurs in pathological situations. In this regard, our group had described GDM in which oxidative stress markers and antioxidants status occurred [23]. Both oxidative and nitrosative stress processes induce specific effects on biological systems, although crosstalk can be observed between them. There is considerable evidence from plants of NO-induced regulation of enzymes associated with oxidative stress [24]. These observations are more limited in mammals. In rat liver, where NO signaling is critical, peroxisome-localized catalase has been identified as one of potential substrates for S-nitrosylation [25]. However, there is a lack of physiological evidence, implicating S-nitrosylated catalase in physiopathological models. NO-mediated activity regulation of other antioxidant enzymes has been described in animal models and includes inhibition of SOD activity associated with increased NO and peroxynitrite in acute coronary syndrome [26], while modulation by S-nitrosylation of peroxiredoxin has been described in cell lines [9]. In GDM placental tissue, S-nitrosylation was not observed in SOD (data no shown). However, it is present in other antioxidant enzymes such as catalase (Figure 2(a)) and peroxiredoxin (Figure 2(b)). A lower activity of catalase has been identified in relation to this posttranslational modification (Figure 2(c)).

Other proteins in which NO-mediated regulation could play a key role under pathological conditions in GDM are those related to placenta cell survival mechanisms and function. MAPK and AKT/PI3-K signaling pathways are crucial in maintaining placental processes by inducing cell replication and trophoblast differentiation [22]. The role of NO in their regulation depends on the specific biological system involved. An example of positive regulation is hyperglycemia-induced nitrosative/oxidative stress which activates ERK1/2 and AKT in glial cells [27]. Conversely, nitrosative stress induces osteoblast apoptosis via MAPK activity [28]. In GDM placental tissue, notably diminished activation levels of ERK1/2 and AKT (Figures 3(a) and 3(c)) are observed, accompanied by increased S-nitrosylation levels in ERK1/2 as well as AKT (Figures 3(b) and 3(d)). These results suggest that nitrosative stress present in GDM induces ERK1/2 and AKT S-nitrosylation which, in turn, causes an impaired activation. These events could explain immaturity features reported by Huynh et al. in GDM placentas [10]. Surprisingly, cytotrophoblast proliferation is increased in GDM placental tissue in association with impaired ERK1/2 activation (Figure 4). This inverse relationship between ERK1/2 activation and trophoblast proliferation has been previously reported [29] and is in accordance with role of ERK1/2 in trophoblast differentiation [30]. Apoptosis-regulating proteins are decisive in pathologic events occurring in placenta during GDM and warrant further study. Caspases are proteases which act as mediators and effectors of apoptosis, and their regulation occurs via different mechanisms including S-nitrosylation [31]. Extensive evidence has been presented describing caspases regulation by the S-nitrosylation process; S-nitrosylation mediated inactivation of caspase-3 is the mechanism by which IL-15 and ghrelin inhibit T cell and gastric mucosal cell apoptosis [32], while caspase-8 is inactivated by S-nitrosylation in NO-mediated rat hepatocyte apoptosis inhibition [33]. In GDM placenta, caspase-3 and caspase-9 present higher activities than healthy controls (Figures 5(a) and 5(b)) and, in the case of caspase-9, a fall in S-nitrosylation level is associated with an increase in cell apoptosis (Figure 5(c)). Observed increment in GDM cytotrophoblasts apoptosis is in line with measured changes in caspase activities and shows the relationship between NO increment, caspase activation, and cytotrophoblast apoptosis in GDM (Figure 5(c)).

## 5. Conclusions

A novel aspect of our findings is that, in placentas from GDM pregnancies, modifications induced by NO in key proteins are associated with pathogenic events. GDM pregnancies represent a proinflammatory status which, potentially, links the future development of metabolic and cardiovascular diseases in both mother and child [34]. The described NO-mediated protein modifications could participate in this process and their presence could be used as biomarkers to predict nitrosative damage and future complications in mother and child.

## Figures and Tables

**Table 1:** Anthropometric and clinical characteristics of GDM and control patients and their offspring (placental samples).

Characteristics	Control (*n* = 8)	GDM (*n* = 8)	*p* value
Delivery mode	Cesarean section	Cesarean section	
Maternal age (years)	30.52 ± 4.5	31.43 ± 4.4	0.4
Parity	1.36 ± 0.7	1.45 ± 0.7	0.6
Gestational age at partum	38.9 ± 1.8	39.2 ± 3.05	0.6
Newborn weight (g)	3270 ± 500	3319 ± 457	0.7
Maternal pregravid BMI (Kg/m^2^)	23.31 ± 4.2	27.13 ± 4.6	0.001
Placental weight (g)	549.2 ± 39.9	612.3 ± 82.6	0.17

Data represent mean ± SEM. BMI: body mass index. ^*∗*^*p* < 0.05 versus control group by Mann–Whitney *U* test.

## Abbreviations

GDM: Gestational diabetes mellitus
iNOS: Inducible nitric oxide synthase
IR: Insulin resistance
ROS: Reactive oxygen species
RNS: Reactive nitrogen species
NO: Nitric oxide
SOD: Superoxide dismutase.
